# The role of sociocultural factors in rare medical conditions: The first case report of pseudocyesis in an Ethiopian woman with major depressive disorder

**DOI:** 10.1002/ccr3.8888

**Published:** 2024-05-07

**Authors:** Faiz Mohammed Kassim, Muluken Tesfaye Wasihun, Meskerem Abebe Jimma, Surafel Worku Megersa

**Affiliations:** ^1^ Department of Psychiatry St. Paul's Hospital Millennium Medical College Addis Ababa Ethiopia

**Keywords:** abdominal distention, depression, ethiopia, false pregnancy, pseudocyesis

## Abstract

Our case report highlights pseudocyesis, a rare medical condition in a 40‐year‐old woman with comorbid major depressive disorder. Cultural influences on experiences, and the need for understanding sociocultural factors in mental health, are emphasized in low‐resource settings.

## 1 INTRODUCTION

A fantasy of pregnancy manifested as pseudocyesis is one of the earliest medical conditions recorded in history, dating back to the time of Hippocrates. Pseudocyesis or false pregnancy is “a false belief of being pregnant that is associated with objective signs and reported symptoms of pregnancy”.^1^ To describe psychopathology, additional terminologies, such as pseudopregnancy, false pregnancy, imaginary pregnancy, simulated pregnancy, spurious pregnancy, or phantom pregnancy, have been used interchangeably.^2^, ^3^ Symptoms that are most frequently observed during pseudocyesis include amenorrhea, morning sickness, abdominal distention, feeling of fetal movement, enlargement of the breasts, areolar pigmentation, secretion of milk, softening of the cervix, lordotic posture on walking or weight gain.^2^, ^4^

The prevalence of pseudocyesis has decreased during the last 70 or 80 years, probably due to a number of sociocultural, family, and medical factors.^5^ It is expected that pseudocyesis is found more commonly in developing countries because of less medical facilities and psychosocial factors.^3^, ^6^, ^7^ However, the case is rarely studied or reported in Africa^8^, ^9^, ^10^, ^11^, ^12^, ^13^ and other developing countries or remote areas. Pseudocyesis is also neglected in Ethiopia. The following pseudocyesis case in a 40‐year‐old woman with major depressive disorder (MDD) helps to discuss the topic.

## 2 CASE HISTORY

This is a 40‐year‐old, Para v abortion II lady who has experienced two induced abortions in the past. She is currently married and lives with her husband and three children. She dropped out of elementary school and is a housewife, relying on her husband as the sole provider for their family, which creates financial difficulties for them. She grew up in a rural area of Adama town in Ethiopia and had a relatively normal childhood without any physical, sexual, or psychological trauma. She got married and converted from Orthodox Christianity to Islam after her husband, which led to her father cursing her. Her first two children died shortly after birth, which she believes was a result of the curse. Due to this experience, she used to deny and pretend not to be pregnant when she actually was. However, she has since given birth to three more children who are alive and healthy. While she still feels sadness about the loss of her first two children, she has a supportive and healthy relationship with her husband.

She is a known patient with a diagnosis of major depressive disorder (MDD) 5 months prior to her current presentation, she was diagnosed after she went to a clinic in Adama with a compliant of tearfulness, depressed mood, loss of interest in almost all activities, difficulty focusing and passive death wish, which lasted for a few months. She was given sertraline 50 mg every morning which progressively escalated to 100 mg every morning, she reported having some improvement with her depressed mood, sleep difficulty, focus, and her death wish.

**FIGURE 1:**
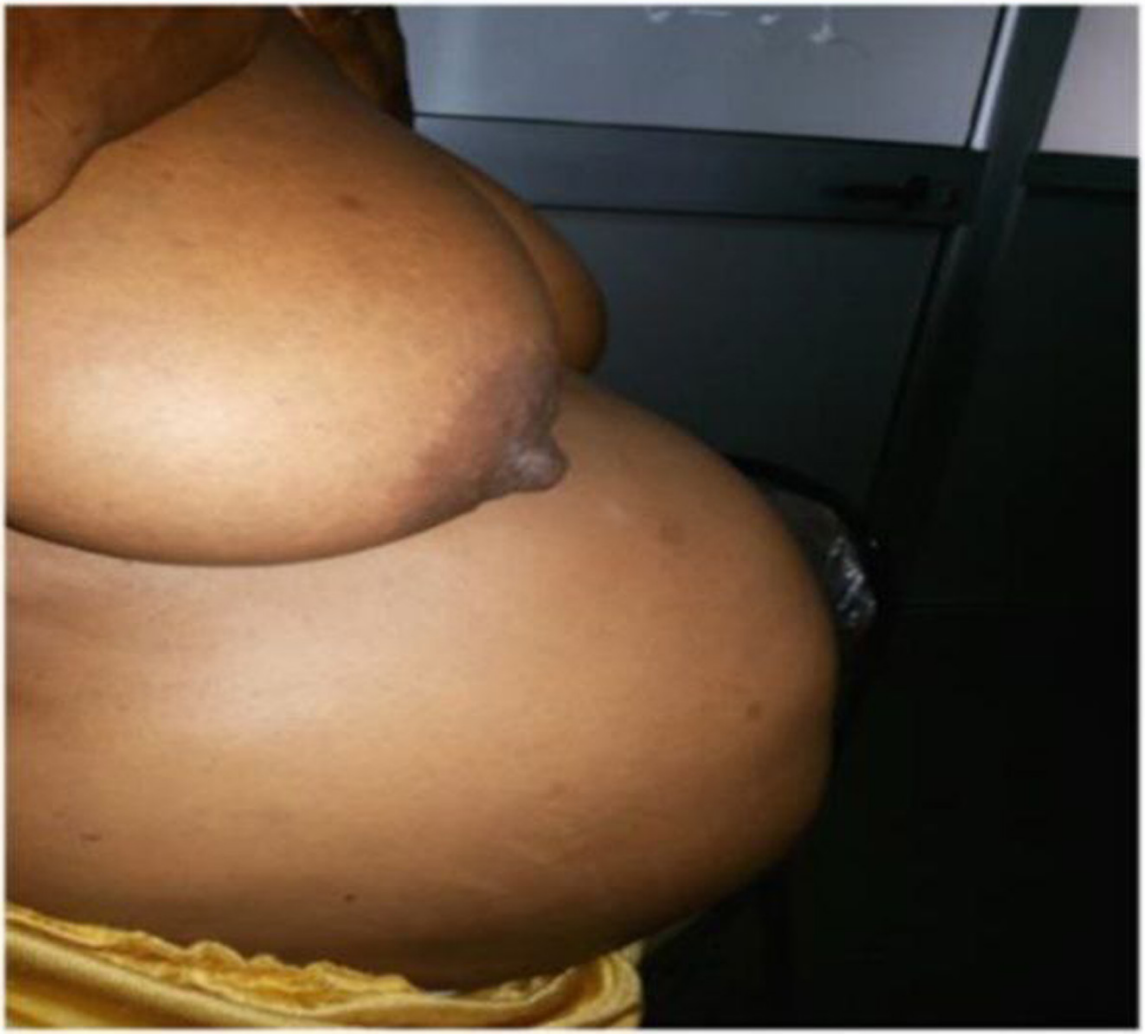
The abdominal and breast picture of the patient, standing position.

**FIGURE 2:**
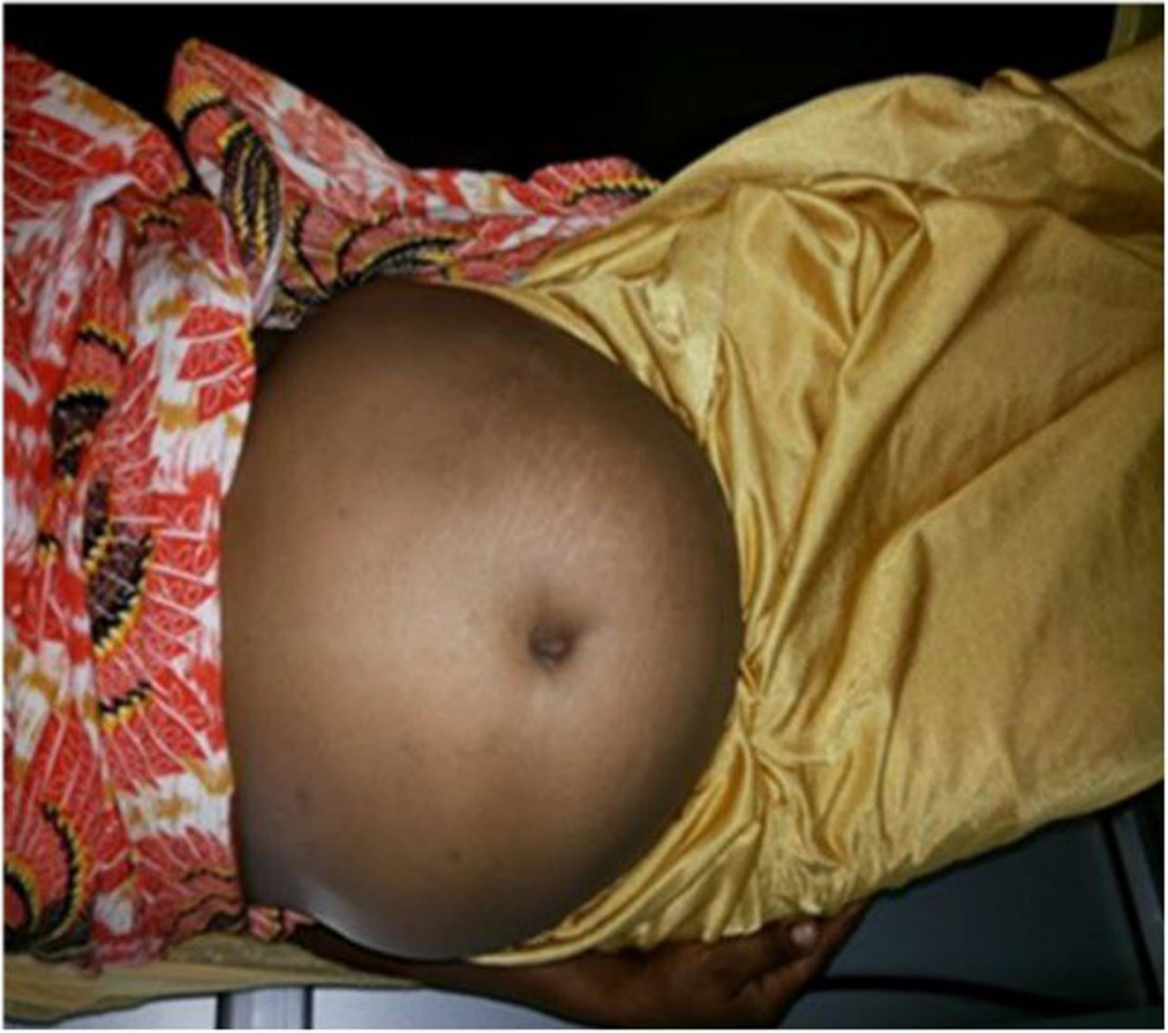
The abdominal picture of the patient, supine position.

Currently, she presented with main compliant of abdominal distension for 4 years. She reported absence of menses occasional experience of nausea and vomiting, mostly occurring during the morning in the first 3–5 months of onset of symptoms 4 years back. The two symptoms were accompanied by abdominal swelling that was initially small in size and then progressively increased to attain its current size (See Figures 1 and 2). She also reported that she has significant weight gain. After 5 months of the onset of the symptoms, she began to feel “fetal kick” on a daily basis and started to have breast engorgement.

She visited more than 18 healthcare facilities (mostly she was covering her medical expenses with the support of her neighbors and relatives) wondering if she was really pregnant or not. Although all of her tests were negative and she was consistently informed that she was not pregnant, she was not convinced. Then, she visited a traditional birth attendant who described the condition as “የአጥንት ልጅ” (in the local language, literally it means “the baby of a bone”). “The baby of a bone” is a cultural explanation for a pregnant woman having vaginal bleeding on multiple occasions, but it was believed that she subsequently could give birth over a duration longer than expected for other pregnancies. Hence, the society gives an explanation that the baby has no blood and is only made of bones.

On mental state exam, she had dysphoric, constricted affect. She was preoccupied with the idea of being pregnant and had no perceptual disturbance. Pertinent physical findings include that she looks older than her stated age. She had symmetrically enlarged breasts, and her abdomen was grossly distended and moved with respiration. She had a lordotic posture. There is moderate abdominal tenderness over the right lower quadrant and there is no palpable mass. There was normoactive bowel sound with no appreciable fetal heartbeat, soft to firm consistency, and no shifting dullness or fluid thrill.

## 3 METHODS

Investigation results of complete blood count, renal, liver, and thyroid function fasting serum glucose, and lipid profile tests were in the normal range. Urine and serum HCG were negative, but serum follicle stimulating hormone (FSH), was three times elevated than the normal lab range. In addition, abdominal ultrasound was unremarkable and did not show the presence of fetus.

With the above history, physical and mental state exam, and considering her investigation results, she was diagnosed to have other specified somatic symptoms‐related disorder (pseudocyesis) + co‐morbid MDD, Delusional disorder (Somatic type)‐Delusion of pregnancy with comorbid depression was also considered for which Olanzapine (5 mg/day, then 10 mg/day) was added. Sertraline was continued with the same dose. She took the olanzapine for 4 months. Supportive psychotherapy was initiated at SPHMMC by her psychiatrist, who is one of the authors of this report.

## 4 OUTCOME AND FOLLOW‐UP

She was followed for a total of 6 months and was showing slight improvement with mostly her depressive symptoms and her coping to the situation, but she was lost to follow up for unexplained reasons.

## 5 DISCUSSION

The aim of the present case report was to demonstrate the impact of sociocultural, economic status, and poor family support on the development of pseudocyesis.

The case highlighted the intricate sociocultural factors that led to the development of pseudocyesis in an adult female with MDD. Studies indicated that most females with pseudocyesis might have mild‐to‐moderate affective disorders, including MDD.^3^, ^5^, ^14^ It has been suggested that depression plays an important role in the etiology of pseudocyesis, as both conditions share some similar pathophysiological mechanisms,^4^ which might explain the present case.

To the best of our knowledge, this is the first recorded case report of pseudocyesis in Ethiopia.

The current patient appears to be grappling with psychological and cultural factors that have led her to have a strong conviction in the “curse” of her father that has persistently stressed her. Her low level of education and unemployment could also be contributing factors. Moreover, low socioeconomic status and poor family support are important factors in the development of pseudocyesis.^15^

Furthermore, pseudocyesis is commonly seen in women who are facing the threat of menopause or who want to preserve their esteem in front of their husbands or their families.^6^ Women with a history of abortion, those who have lost one or more children due to death, or those who excessively fear losing their children in the future are also at a higher risk for pseudocyesis.^5^, ^16^, ^17^ These factors provide some insight into the psychological state of our patients.

Consistent with previous reports, the current patient exhibited symptoms such as amenorrhea, nausea, vomiting, abdominal distention (See Figures 1 and 2), enlargement of the breasts (see Figure 1), areolar pigmentation, lordotic posture on walking, and weight gain.^2^, ^4^ However, laboratory tests (i.e., urine and serum HCG) were negative, and abdominal ultrasound did not support the presence of pregnancy, and the diagnosis of pseudocyesis was. As all or most patients would have the difficulty of accepting the objective laboratory results/reality,^7^ our current patient was visiting dozens of clinics wishing to hear the news that “she is pregnant!” We provided psycho‐education and support to help the patient recognize the condition and accept her condition, but unfortunately, she was lost to follow up after a few months.

Our patient was a 40‐year‐old woman presenting with galactorrhea. It is noteworthy that, a diagnosis of pseudocyesis without galactorrhea, may be more common in older than younger women.^18^ Pseudocyesis commonly occurs in females who are in the reproductive age group of 20–44,^17^ mainly in those in their late 30s. However, there are some reports that the problem has been observed in female teenagers^18^, ^19^, ^20^, ^21^, ^22^, ^23^ and, surprisingly, in preteen^24^ and 6‐year‐old girls.^14^, ^25^

The case has also been observed in males,^26^, ^27^, ^28^, ^29^ although most of the cases in males would not be categorized under pseudocyesis based on the latest standards.^5^

The pathophysiology of pseudocyesis remains largely unknown, but it is suggested that the complex interactions of psychological, sociocultural, and endocrine factors may be crucial.^4^, ^5^, ^6^, ^7^, ^17^, ^19^ Psychological and/or social factors may impact the hypothalamic–pituitary–ovarian, and it has been suggested that endocrine traits may serve as a linking pathway between pseudocyesis pathophysiology and MDD.^3^ Stress or the excess desire for pregnancy could stimulate the release of pituitary hormones, such as gonadotropin‐releasing hormone (GnRH).^16^ The increased level of FSH in the present patient or other patients might be because of reduced steroid‐dependent negative feedback mechanisms on GnRH.^3^, ^4^ Importantly, dysregulation of monoaminergic pathway in the central nervous system causing a deficit in dopamine and noradrenaline, and increased autonomic activities because of noradrenaline turnover may play a key role (see 3 for review).

Our patient was not rightly diagnosed, despite visiting several healthcare facilities, creating awareness among clinicians might contribute to early detection and better outcomes, and we believe that our report sheds light on the role of socioeconomic factors in the development of pseudocyesis and highlights the need for interventions to address these factors. We suggest a collaborative approach of obstetrics/gynecologists and psychiatrists/mental health professionals in the diagnosis and management of pseudocyesis with or without other comorbid disorders. Furthermore, the present case has important implications for subsequent quantitative and qualitative studies regarding the development of pseudocyesis and for the development of interventions and policies aimed at reducing the incidence of pseudocyesis, particularly in low‐income and marginalized populations.

Overall, this case study underscores the importance of considering the sociocultural context in the diagnosis and treatment of rare medical conditions and highlights the potential benefits of a culturally sensitive approach to mental health care. Considering our observation, we suggest that future studies should focus on the impact of sociocultural factors, as well as other comorbid psychiatric conditions, and the development of effective preventive and intervention strategies for pseudocyesis.

## AUTHOR CONTRIBUTIONS

**Faiz Mohammed Kassim:** Conceptualization; methodology; supervision; validation; writing – original draft; writing – review and editing. **Muluken Tesfaye Wasihun:** Conceptualization; methodology; writing – review and editing. **Meskerem Abebe Jimma:** Methodology; writing – review and editing. **Surafel Worku Megersa:** Conceptualization; methodology; supervision; validation; writing – original draft; writing – review and editing.

## FUNDING INFORMATION

There was no available funding for the current study.

## CONFLICT OF INTEREST STATEMENT

The authors declare that there are no conflicts of interest described in this case report.

## CONSENT

Written informed consent was obtained from the patient for publication of this case report.

## Data Availability

Not applicable.
